# Extracranial Metastases in Glioblastoma, IDH-Wildtype: A Case Series

**DOI:** 10.3390/diagnostics16071094

**Published:** 2026-04-05

**Authors:** Valèria Richart, Marta García de Herreros, Juan Andrés Mora, Camilo Pineda, Iban Aldecoa, Estela Pineda, Izaskun Valduvieco, José Juan González, Laura Oleaga, Sofía González-Ortiz

**Affiliations:** 1 Department of Radiology, Hospital Clinic de Barcelona, 08036 Barcelona, Spain; jamoras@clinic.cat (J.A.M.); cpineda@clinic.cat (C.P.); loleaga@clinic.cat (L.O.); sgonzal@clinic.cat (S.G.-O.); 2 Department of Oncology, Hospital Clinic de Barcelona, 08036 Barcelona, Spain; garciadehe@clinic.cat (M.G.d.H.); epineda@clinic.cat (E.P.); 3 Department of Pathology, Biomedical Diagnostic Center (CDB), Hospital Clinic de Barcelona, University of Barcelona, and Neurological Tissue Bank of the Biobank, FCRB/IDIBAPS, 08036 Barcelona, Spain; ialdecoa@clinic.cat; 4 Radiotherapy Oncology Service, Hospital Clinic de Barcelona, 08036 Barcelona, Spain; ivalduvi@clinic.cat; 5 Department of Neurosurgery, Hospital Clinic de Barcelona, 08036 Barcelona, Spain; jjgonzal@clinic.cat

**Keywords:** glioblastoma, extracranial metastasis, neuro-oncology, molecular profile

## Abstract

**Background**: Extracranial metastasis (EM) from glioblastoma (GB), IDH-wildtype (WHO CNS 2021 grade 4) is rare and often under-recognized, yet it has immediate implications for staging and management. We report a case series integrating advanced neuroimaging, whole-body imaging, and pathology/biomarkers to characterize imaging–pathology correlates of EM and highlight practical clinical triggers that should prompt systemic evaluation. **Case presentation**: We report three patients with adult-type, IDH-wildtype GB who developed EM confirmed by cytology/histology and/or concordant multimodality imaging. Brain MRI (1.5T/3T) demonstrated aggressive primary tumors with qualitative elevation of DSC-perfusion and frequent tumor–surface contact (dural, ependymal/leptomeningeal contact). Intratumoral susceptibility signal reached grade 3 where assessed. All patients underwent surgical resection followed by temozolomide-based chemoradiation; two received fotemustine and bevacizumab, and one underwent re-irradiation. EM presented with clinical triggers including severe axial/back pain, palpable cervical masses, and/or cytopenias. Initial EM sites were bone marrow/vertebrae (*n* = 1) and cervical lymph nodes (*n* = 2); staging revealed additional osseous disease in both nodal cases and a small pulmonary nodule in one. Nodal and osseous lesions were FDG-avid on 18F-FDG PET/CT. OLIG2-positive cytology confirmed cervical nodal metastases, and bone marrow aspiration with GFAP/OLIG2 positivity confirmed medullary infiltration. All tumors shared a molecular profile of TERT-promoter mutation, ATRX wild-type, TP53 mutation, and MGMT-promoter methylation. Despite attempts at second- and third-line therapies, disease progression was rapid, and all patients succumbed within 8–16 months of diagnosis. **Discussion**: This series underscores that EM can occur despite MGMT-promoter methylation and supports the concept of heterogeneous metastatic phenotypes in GB. Our cases reinforce that new axial/back pain or hematologic abnormalities may signal osseous or marrow involvement, and necrotic cervical lymphadenopathy in GB patients warrants dedicated imaging and tissue confirmation with glial markers. Integrating brain MRI features (high perfusion, surface contact, susceptibility burden) with FDG-PET/CT and targeted cytology/pathology can expedite diagnosis and inform multidisciplinary care. **Conclusions**: EM can arise despite MGMT-promoter methylation in IDH-wildtype GBM. Imaging red flags (high perfusion, surface contact, necrotic/FDG-avid cervical nodes) and clinical cues (axial pain, cytopenias, neck masses) should prompt early systemic staging (CT/PET-CT) and targeted tissue confirmation to advance management.

## 1. Introduction

Glioblastoma (GBM) is the most common primary malignant tumor of the adult central nervous system. In the 2021 World Health Organization (WHO) classification, GBM corresponds to an adult-type diffuse glioma, IDH-wildtype, designated WHO grade 4 based on necrosis and/or microvascular proliferation [1,2,3]. Standard care comprises maximal safe resection followed by radiotherapy with concomitant and adjuvant temozolomide, yet median overall survival remains approximately 15 months [4].

Extracranial metastases (EM) of GBM are uncommon, reported in 0.4–2% of patients [5,6,7]. Proposed explanations for the historical rarity include limited survival, the protective role of the blood–brain barrier, and the absence of a permissive extracranial microenvironment [8,9,10,11,12]. Notably, the presence of EM has not consistently been associated with worse overall survival than non-metastatic GBM, and age has not emerged as a clear prognostic factor [6,13]. When EM occurs, the most frequently involved sites include bone (particularly vertebrae and pelvis), cervical lymph nodes, and lung [8,13,14]. Recent reports suggest the true incidence may be higher than historically appreciated, in part due to improved survival, evolving therapies, and heightened clinical awareness [13,15,16].

In this case series, we report three patients with GB who developed histologically confirmed extracranial metastases, involving bone, cervical lymph nodes, and lung. We specifically highlight their molecular profiles, radiological characteristics, and potential factors that may have contributed to metastatic spread. The three patients included in this series were consecutive cases of glioblastoma with extracranial metastases identified between 2019 and 2022 through review of cases presented at our institutional Neuro-Oncology Committee.

## 2. Case Presentation

**Figure 1:**
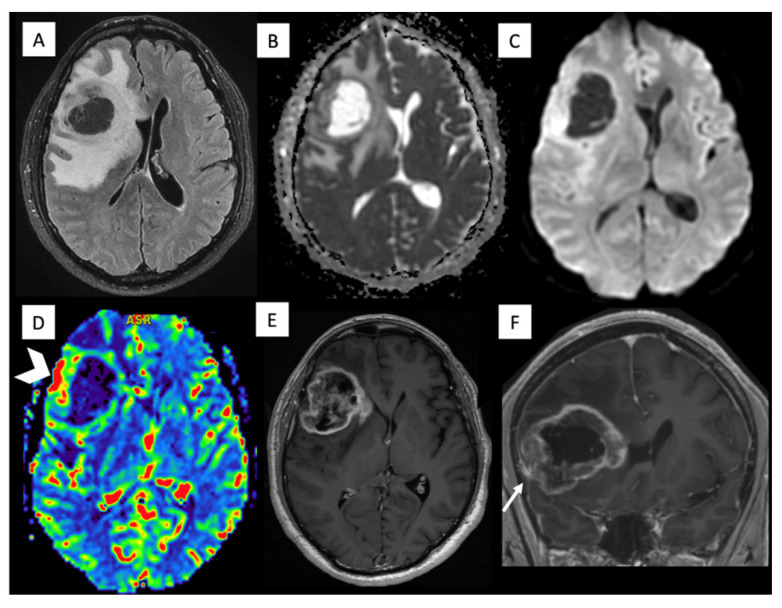
Brain MRI of Patient 1. Brain MRI axial fluid-attenuated inversion recovery (FLAIR) (**A**), ADC (**B**), diffusion b1000 (**C**), DSC perfusion (**D**) and axial and coronal contrast-enhanced T1-weighted (**E**,**F**) images show a right frontal lobe mass with a necrotic center and peripheral enhancement, edema, restricted diffusion, increased rCBV (arrowhead), and meningeal extension (arrow).

**Figure 2:**
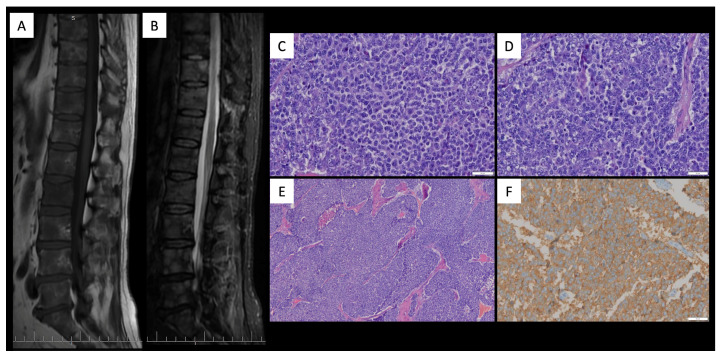
Spinal MRI and bone marrow aspiration of the Patient 1. Sagittal spinal MRI T1-weighted (**A**) and short tau inversion recovery (STIR) (**B**) images show multifocal infiltration of the bone. Bone marrow aspiration (**C**–**E**) hematoxylin-eosin staining and (**F**) synaptophysin staining show a practical absence of normal hematopoiesis, which is displaced by marked infiltration of atypical cells, suggesting bone marrow infiltration by metastases from glioblastoma.

Case 1:

A middle-aged male with no significant medical history presented to the emergency department with a two-month history of persistent headaches and new-onset left hemiparesis. Brain MRI showed a right frontal mass with avid irregular enhancement, central necrosis, superficial meningeal contact, foci of diffusion restriction, and elevated relative cerebral blood volume (rCBV) (Figure 1). Partial resection confirmed GBM, IDH-wildtype with microvascular proliferation, and the molecular profile demonstrated TP53 mutation, ATRX wild-type, TERT-promoter mutation, MGMT-promoter hypermethylation, and EGFR positivity. He received treatment that included chemoradiation with temozolamide. Despite initial treatments, follow-up brain MRI showed disease progression, revealing areas with contrast enhancement and increased rCBV, indicating regions of high vascularity and potential tumor activity. He consequently started a second-line treatment with fotemustine. Subsequent follow-up brain MRI showed disease progression and cerebral edema, prompting the initiation of antiangiogenic treatment with bevacizumab. This treatment yielded a radiological positive response to treatment after two months, as follow-up MRI showed a reduction in edema and mass effect, as well as a decrease in the size of the contrast-enhancing area. Concomitantly, the patient’s condition worsened with new symptoms of lumbar pain. Spinal MRI (Figure 2) revealed multiple bone metastases within vertebral bodies, and concurrent laboratory tests showed pancytopenia and thrombocytopenia. A bone marrow aspiration was performed (Figure 2), which confirmed glial (GFAP/OLIG2-positive) metastasis. A follow-up thoracoabdominal CT found no additional sites. He transitioned to palliative care and died 16 months after diagnosis.

**Figure 3:**
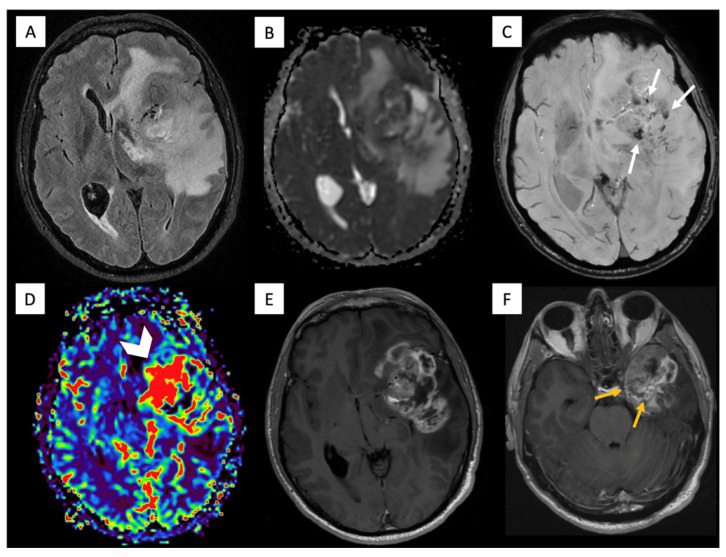
Brain MRI of Patient 2. Brain MRI axial FLAIR (**A**), ADC (**B**), T2 star-weighted angiography (SWAN) (**C**), DSC perfusion (**D**), and contrast-enhanced T1-weighted (**E**,**F**) images show a frontotemporal lobe mass with a necrotic center and peripheral enhancement, edema, high-grade ITSS (white arrows), and increased rCBV (arrowhead). Dural extension and ependymal infiltration of the temporal horn are also noted (yellow arrows).

**Figure 4:**
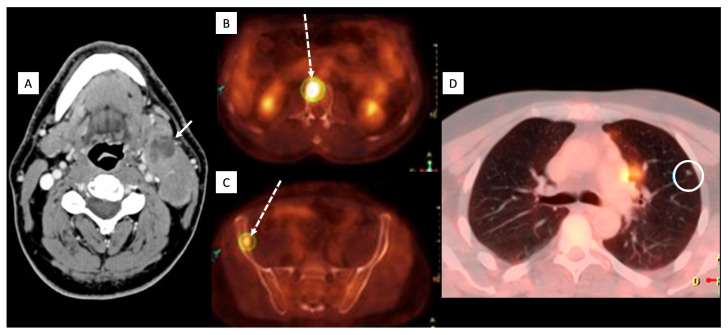
Neck CT and body PET-CT of Patient 2. Axial contrast-enhanced neck CT (**A**) shows left laterocervical necrotic lymph nodes (arrow). Body PET/CT (**B**–**D**) shows increased FDG uptake in lumbar body vertebra, right iliac wing (dashed arrows), and a small pulmonary nodule (circle), consistent with metastases.

Case 2:

A middle-aged male with no relevant prior medical history presented to the emergency department with persistent headaches and dysphasia. Brain MRI demonstrated a left frontotemporal mass with heterogeneous enhancement, central necrosis, hemorrhagic foci with high intratumoral susceptibility signal (ITSS) (grade 3), elevated rCBV, and dural/ependymal extension (Figure 3). The patient underwent a partial resection. Pathology showed GBM with microvascular proliferation and necrosis; immunohistochemistry revealed GFAP-positive neoplastic cells, focal TP53 positivity, EGFR overexpression, and ATRX/IDH1 wild-type. Sequencing confirmed TERT-promoter mutation, MGMT hypermethylation, and no BRAF mutation. During chemoradiation with temozolamide, a palpable laterocervical mass appeared, and neck CT showed lymphadenopathy. Ultrasound fine needle aspiration (US-FNA) confirmed OLIG2-positive metastasis, and 18F-FDG PET/CT demonstrated additional bone disease and a small lung nodule (Figure 4). He started a second-line treatment with fotemustine but showed no clinical benefit. Consequently, a third-line treatment with antiangiogenic bevacizumab was initiated but his condition rapidly deteriorated, preventing further oncological interventions. He died eight months after initial diagnosis.

**Figure 5:**
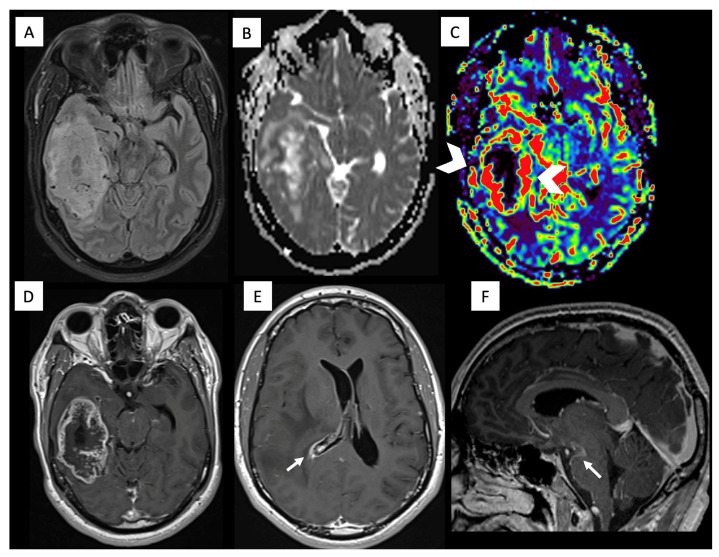
Brain MRI of Patient 3. Brain MRI axial FLAIR (**A**), ADC (**B**), DSC perfusion (**C**), and axial and sagittal contrast-enhanced T1-weighted (**D**–**F**) images show a temporal lobe mass with a necrotic center and peripheral enhancement, edema, and increased rCBV (arrowheads). Ependymal extension of the right ventricle and leptomeningeal extension are also noted (arrows).

**Figure 6:**
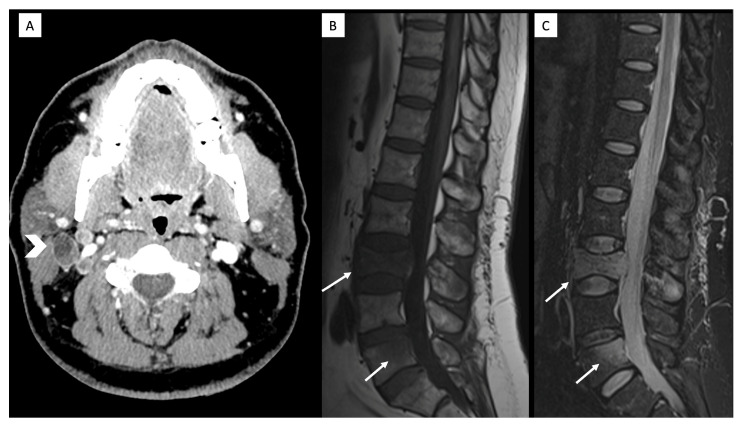
Neck CT and spinal MRI of Patient 3. Axial contrast-enhanced neck CT (**A**) shows multiple necrotic right cervical lymph nodes (arrowhead). Sagittal spinal MRI T1-weighted (**B**) and STIR (**C**) images show metastatic infiltration of L3 and L5 (arrows) and a pathological fracture of L3.

Case 3:

A middle-aged male with no significant medical history presented to the emergency department with persistent headaches and seizures. Brain MRI showed a right temporal infiltrative mass with irregular solid enhancement, necrotic component, peripheral elevated rCBV, foci of hemorrhage, and ependymal/leptomeningeal extension (Figure 5). The patient underwent partial resection. Pathology report confirmed GBM with sarcomatous change and leptomeningeal infiltration; the tumor was IDH-wildtype, TP53-mutated, ATRX wild-type, TERT promoter-mutant, MGMT-methylated, and had no BRAF mutation. He underwent standard chemoradiotherapy with temozolomide. Despite initial treatment, the patient developed severe back pain and was hospitalized requiring high doses of opioids. Spinal MRI showed multiple bone metastases with pathological fractures, as well as enlarged lymph nodes, and additional imaging studies with neck CT showed necrotic laterocervical lymphadenopathy (Figure 6). US-FNA confirmed OLIG2-positive nodal metastasis. A follow-up 18F-FDG PET/CT confirmed previously known cervical lymphadenopathy and osseous metastases, without evidence of new lesions. He transitioned to palliative care due to his declining condition and passed away approximately thirteen months after initial diagnosis.

**Figure 7:**
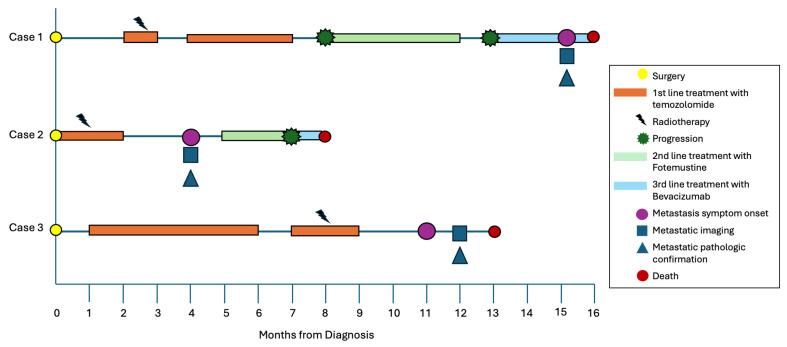
Swimmer plot showing the clinical timeline of three patients with glioblastoma and extracranial metastases. Each horizontal line represents one patient. Time is shown in months from initial diagnosis. Thick bars indicate periods of systemic treatment. Symbols represent key clinical events, including surgery, disease progression, onset and confirmation of extracranial metastases, and death.

In all cases, death was attributed to progression of the primary intracranial glioblastoma rather than extracranial metastatic disease. The clinical course of each patient is summarized in a swimmer-style timeline (Figure 7). Table 1 summarizes presentation, molecular profile, and key MRI features, and Table 2 details extracranial metastases and survival intervals.

## 3. Discussion

GBM is known for its aggressive nature and poor prognosis, yet the development of metastasis outside the central nervous system (CNS) is a rare occurrence. In this study, bone (especially vertebrae and pelvis), cervical lymph nodes, and lungs emerged as the most frequent sites of extraneural disease, in keeping with the previous literature [8,13,14].

All three tumors shared a consistent molecular profile (IDH-wildtype, MGMT-promoter-methylated, TERT-promoter-mutant, ATRX wild-type, and TP53-mutation). While MGMT methylation generally predicts sensitivity to alkylating therapy and longer survival, it does not exclude metastatic invasion. In contrast, an osseous-metastasis case series [15] reported that all metastatic GBMs were MGMT promoter-unmethylated, and that most of them harbored TP53 pathway alterations, suggesting a more aggressive, chemotherapy-resistant, metastasis-prone biological phenotype [15,17]. A comparative analysis likewise found decreased MGMT methylation and more frequent TP53 mutations among GBMs with extracranial spread versus those without [18]. In addition, sarcomatous dedifferentiation, a histologic variant present in one of our cases, has been associated with higher metastatic potential [19]. This finding also appeared in a recent single-center series, in which a subset of patients displayed gliosarcomatous features [15]. These data support a polygenic, phenotype-level model in which MGMT status alone is insufficient for risk stratification; however, co-alterations such as TP53 and sarcomatous transformation may be more proximate drivers of an extracranial-prone phenotype. This interpretation should be tempered by legacy taxonomy effects. Before WHO 2021, some IDH-mutant tumors were labeled “GBM”, and some systematic reviews of extracranial metastases included IDH-mutant cases, so apparent molecular patterns across eras may be confounded [15].

Extraneural dissemination of GBM is likely multifactorial. Proposed routes include hematogenous spread, facilitated by tumor angiogenesis or iatrogenic disruption after craniotomy/biopsy/shunting, lymphatic pathway via the meningeal–glymphatic network into dural lymphatics and onward to deep cervical nodes, and direct extension through skull, foramina, or perineural pathways [11,12,15,16,19]. Although invasive procedures may create conduits [19,20], documented metastases in patients without prior surgery and comparable circulating tumor cell levels before and after resection suggest GBM cells can breach the blood–brain barrier (BBB) independently [11,21]. Our cohort reflects both patterns, as two patients developed cervical nodal disease consistent with meningeal lymphatic drainage, and one had vertebral/bone marrow involvement indicated by anemia and thrombocytopenia and confirmed on aspiration. These abnormal analytic values can hint at medullary infiltration, as seen in other published cases [22,23]. Bone tropism may be supported by GBM expression of hematopoietic stem cell programs and pro-angiogenic cues that favor survival within the marrow niche 19,26. Overall, these observations support concurrent hematogenous and lymphatic routes and indicate that surgical history is neither necessary nor sufficient for metastasis, favoring early use for systemic imaging when new axial pain, cytopenias, or cervical lymphadenopathy arise.

All our patients demonstrated brain MRI findings indicative of high-grade gliomas, including contrast enhancement, necrosis, increased rCBV on dynamic susceptibility-weighted contrast-enhanced (DSC) MR perfusion, and low apparent diffusion coefficient (ADC) values on diffusion. These features are hallmarks of glioblastoma and reflect increased tumor cellularity, angiogenesis and BBB disruption. Diffusion and ADC values in gliomas reflect microscopic architecture, in particular, low ADC values in the solid tumor components correlate with high cellular density [24]. In addition, angiogenesis and neovascularity are associated with tumoral viability and aggressiveness, reflected by ITSS and rCBV. High-grade ITSS, assessed on susceptibility-weighted images (SWI) in case 2, appeared as prominent dotlike or fine linear hypointensities, corresponding to intratumoral microhemorrhage and neoangiogenesis. This finding has been shown to correlate with both histologic tumor grade and elevated CBV on MR perfusion [25]. All our patients underwent DSC MR perfusion, revealing qualitative increased rCBV in comparison with contralateral white matter, especially in the solid-enhancing tumor component, which significantly correlates with glioma grading as determined by histopathology [26].

Furthermore, dural extension, ependymal spread, and leptomeningeal infiltration were observed in our patients, seen as tumor abutment or involvement of the dural or ependymal surfaces on MRI, with pathological confirmation of leptomeningeal infiltration in case 3. This infiltration may be associated with potential access to meningeal lymphatic channels and deep cervical lymph nodes, as described by others [12,16,27,28], as well as hematogenous spread. Although such radiological signs illustrate the aggressive and infiltrative nature of these tumors, they may support consideration of more vigilant systemic surveillance, especially in patients with concerning clinical or laboratory findings.

Salvage treatments for recurrent GBM typically involve nitrosoureas, such as fotemustine, and anti-VEGF therapies like bevacizumab. Current research trends reflect a significant shift toward combined molecular targeted therapies rather than monotherapies to better combat drug resistance. Much of this research focuses on the protein kinase and microenvironmental pathways, targeting key entities such as EGFR and VEGF [29]. While bevacizumab is a common salvage agent, its clinical impact is often inconsistent due to tumor heterogeneity, which may contribute to variability in treatment response. Furthermore, some studies have reported that suppression of VEGF signaling may be associated with pro-invasive response in GBM cells [11,15], likely mediated by the Src family kinase pathway. To address this, contemporary studies explore combining bevacizumab with Src inhibitors to block treatment-induced migration and invasion [29]. Given the extracranial spread observed in our patients, our observations remain descriptive, and no causal relationship between specific therapies and metastatic behavior can be established. Nevertheless, documenting the timing of treatments in relation to disease evolution may help to better contextualize these findings.

Routine systemic staging is not recommended given the rarity of extracranial spread in GBM [30]; however, several clinical triggers should lower the threshold for body imaging. New or disproportionate axial/back pain warrants evaluation because osseous metastasis, predominantly vertebral, is the most frequent extracranial site and often declares itself around a year from diagnosis (median ~12–13 months), with very short survival once detected. Unexplained cytopenias/pancytopenia should prompt consideration of bone marrow involvement. Palpable cervical lymphadenopathy merits targeted imaging because cervical/parotid nodes from GBM are typically FDG-avid on PET/CT and centrally necrotic/peripherally enhancing on contrast CT, and cytology can confirm glial origin. In these scenarios, contrast-enhanced CT of the neck/chest and/or 18F-FDG PET/CT are appropriate first-line studies, with US-guided FNA (including OLIG2/GFAP immunocytochemistry) for nodal confirmation. In order to facilitate early recognition of extracranial disease, we propose a pragmatic approach linking common clinical presentations with appropriate first-line investigations (Table 3).

## 4. Conclusions

In summary, this case series illustrates that GBM, despite its traditionally intracranial nature, can spread outside the CNS—especially to bone and lymph nodes—underlining the complexity of tumor biology and the importance of vigilant clinical assessment. Molecular indicators, such as TP53 mutation or gliosarcoma histology, and radiological features, such as dural extension, leptomeningeal dissemination, and marked neovascularization (e.g., high ITSS, elevated rCBV), may signal a need for vigilance regarding extracranial disease. As the underlying mechanisms remain elusive, further research is essential. Incorporating genomic profiling, advanced imaging techniques, and prospective data collection could clarify the metastatic pathways of GBM. This enhanced understanding is crucial to developing targeted surveillance strategies and, ultimately, more effective treatment protocols for this aggressive malignancy.

## Figures and Tables

**Table 1:** Clinical, radiological and molecular characteristics of the three cases.

Feature	Case 1	Case 2	Case 3
**Sex**	M	M	M
**Clinical Presentation**	Headache, hemiparesis	Headache, speech disturbance	Headache, seizures
**Tumor Location**	Right frontal	Left frontotemporal	Right temporal
**Surgery**	Partial resection	Partial resection	Partial resection
**Key MRI Findings**	Increased rCBV Dural extension	Increased rCBV Increased ITSS Dural and leptomeningeal extension	Increased rCBV Ependymal and leptomeningeal extension
**Histopathology And Molecular Features**			
*Histological Diagnosis*	Glioblastoma, WHO grade 4	Glioblastoma, WHO grade 4	Glioblastoma, WHO grade 4
*IDH1/IDH2 Status*	Wildtype	Wildtype	Wildtype
*TERT-Promoter Mutation*	Mutated	Mutated	Mutated
*MGMT-Promoter Methylation*	Methylated	Methylated	Methylated
*ATRX Status*	Wildtype (retained)	Wildtype (retained)	Wildtype (retained)
*P53 Status*	Mutated	Mutated	Mutated
*EGFR Status*	Positive	Positive	Not performed
*GFAP Expression*	Positive (metastasis)	Positive	Not performed
*OLIG2 Expression*	Positive (metastasis)	Positive (metastasis)	Positive (metastasis)
*BRAF Mutation*	Not performed	Not detected	Not detected

Data are shown per patient. M = male. Tumor site = dominant lobe/region at diagnosis. Key MRI findings include dural/ependymal/leptomeningeal contact, qualitative DSC-perfusion (normalized rCBV elevated; ratios not available), and ITSS, where assessed. Surgery = extent (maximal/partial).

**Table 2:** Metastatic presentation triggers, sites, proof, and survival intervals.

Case	Presentation/Trigger	First EM Site(s) & Proof	Additional Sites at Staging	Time to EM (Month)	EM → Death (Month)	OS (Month)
1	Severe low back pain; cytopenias	Bone marrow/vertebrae; marrow aspiration GFAP/OLIG2+	Body CT: none	15.0	1.0	16.0
2	Palpable laterocervical mass; back pain	Cervical nodes; US-FNA OLIG2+	FDG PET/CT: bone (vertebrae, right pelvis) and small lung nodule	4.0	4.0	8.0
3	Severe low back pain	Right cervical nodes & lumbar vertebrae; US-FNA OLIG2+; MRI/PET-CT concordant	FDG PET/CT: none	12.0	1.0	13.0

For each patient, the presentation trigger indicates the clinical sign/symptom or laboratory abnormality prompting systemic work-up. First EM sites and proof lists the initial extracranial site(s) with method of confirmation (e.g., US-FNA with OLIG2/GFAP immunocytochemistry; bone-marrow aspiration with glial markers; or unequivocal CT/PET-CT/MRI when tissue was not obtained). Additional sites at staging reflect lesions identified on subsequent systemic imaging. Time to EM is the interval from GBM diagnosis to first EM (months). EM → death is the interval from first EM to death (months). OS is overall survival from GBM diagnosis to death (months). Values are reported to one decimal place when applicable.

**Table 3:** Suggested initial diagnostic approach based on clinical triggers of suspected extracranial metastasis in glioblastoma.

Clinical Presentation	Suspected Site of Metastasis	Recommended First-Line Evaluation
Persistent axial or back pain	Bone or vertebral metastasis	MRI spine; consider CT if MRI unavailable
Cytopenias (e.g., anemia, thrombocytopenia)	Bone marrow infiltration	Complete blood count, peripheral smear, bone marrow aspiration
Palpable cervical or neck mass	Lymph node metastasis	Neck CT and/or ultrasound with fine needle aspiration
Unexplained systemic symptoms (e.g., fever, weight loss)	Systemic dissemination	Thoracoabdominal CT or PET-CT
Respiratory symptoms or incidental pulmonary findings	lung metastasis	Chest CT; consider PET-CT for staging

## Data Availability

All de-identified data supporting the findings of this study are included within the article and its additional files; further materials are available from the corresponding author on reasonable request.

## Abbreviations

The following abbreviations are used in this manuscript:

GBM	Glioblastoma
WHO	World Health Organization
EM	Extracranial metastasis
rCBV	Relative cerebral blood volume
ITSS	Intratumoral susceptibility signal
US	Ultrasound
FNA	Fine needle aspiration
CNS	Central nervous system
BBB	Blood–brain barrier
DSC	Dynamic susceptibility-weighted contrast-enhanced
ADC	Apparent diffusion coefficient
SWI	Susceptibility-weighted images
